# Vitamin D receptor gene polymorphisms in multiple sclerosis patients in northwest Greece

**DOI:** 10.1186/1477-5751-10-3

**Published:** 2011-05-05

**Authors:** Chrissa Sioka, Stylianos Papakonstantinou, Sofia Markoula, Foteini Gkartziou, Athanasia Georgiou, Ioannis Georgiou, Sygliti-Henrietta Pelidou, Athanassios P Kyritsis, Andreas Fotopoulos

**Affiliations:** 1 Department of Nuclear Medicine, University Hospital of Ioannina, Ioannina, Greece; 2 Department of Neurology, University Hospital of Ioannina, Ioannina, Greece; 3 Neurosurgical Research Institute, University of Ioannina, Ioannina, Greece; 4 Laboratory of Medical Genetics, University Hospital of Ioannina, Greece

## Abstract

**Background:**

Polymorphisms of the vitamin D receptor (VDR) gene have been linked to both multiple sclerosis (MS) and osteoporosis. We examined the frequency of the Taq-I and Bsm-I polymorphisms of the vitamin D receptor (VDR) gene in 69 patients with MS and 81 age and sex-matched healthy individuals. Genotyping of Taq-I (rs731236) and Bsm-I (rs1544410) was performed using TaqMan^® ^SNP Genotyping Assay. All patients and controls had determination of body mass index (BMI), bone mineral density (BMD) and smoking history.

**Results:**

The mean age of patients was 39 ± 10.5 years compared to 38.7 ± 10.7 years of the controls (p = 0.86), the BMI was 24.8 ± 4.2 kg/m^2 ^compared to 25.7 ± 4.8 kg/m^2 ^of the controls (p = 0.23), the BMD in the lumbar spine 0.981 ± 0.15 compared to 1.025 ± 013 of the controls (p = 0.06) and the total hip BMD was 0.875 ± 0.14 compared to 0.969 ± 0.12 of the controls (p < 0.001). There were no differences of the Taq-I (TT, CT, CC) and Bsm-I genotypes (GG, GA, AA) and allelic frequencies between MS and control individuals. Multivariate analysis also failed to show any association of the Taq-I and Bsm-I polymorphisms and MS or sex, BMI, BMD and smoking history.

**Conclusions:**

This study suggests that the Taq-I and Bsm-I polymorphisms of the VDR gene are not associated with MS risk, BMI or BMD in the Greek population studied.

## Background

Multiple sclerosis (MS) is a progressive demyelinating disease of the central nervous system (CNS) that occurs mainly in young adults, frequently leading to substantial disability several years after diagnosis [1]. Vitamin D is essential for bone and mineral homeostasis and exhibits immunoregulatory and anti-inflammatory properties [2]. Abnormal regulation of vitamin D metabolism has been linked to MS and other diseases such as type 1 diabetes, cancer, and osteoporosis [3-7]. Vitamin D acts through the vitamin D receptor (VDR) and the membrane associated rapid response steroid binding receptor (MARRS) [8]. Certain polymorphisms of the VDR gene may modify vitamin D function and metabolism and have been examined in studies evaluating the role of vitamin D on MS [9].

Several such studies that have investigated the role of VDR gene polymorphisms in various ethnic groups have produced conflicting results. A Japanese study reported an association between the Bsm-I VDR gene allelic polymorphism and MS [10]. An Australian MS case-control study found a significant difference of the Taq-I polymorphism genotype distribution in MS patients [11]. Similar results were reported in other studies [12]. Nevertheless, other studies found no differences of a number of VDR gene polymorphisms between MS and control groups [13-15]. However, these as well as other studies reported other associations of the VDR gene polymorphisms, such as a significant interaction between winter sun exposure during childhood [13], or interaction between dietary intake of vitamin D and VDR gene polymorphisms on MS risk [16], and involvement of the Fok-I VDR gene polymorphism in vitamin D metabolism [14]. In the present study we investigated the Taq-I and Bsm-I polymorphisms of the VDR gene in a cohort of MS patients and a control group in Northwest Greece.

## Methods

### Patients

The individuals recruited in this study were 69 consecutive MS patients referred to the Nuclear Medicine Department for spinal and femoral BMD measurements, as part of a screening program of all MS patients for osteopenia or osteoporosis, and 81 control individuals matched for sex and age. All patients satisfied the Mc Donald criteria for MS diagnosis [17]. None of the case and control individuals were receiving chronically steroids, bisphosphonates or selective estrogen receptor modulators. Control individuals consisted of non-family visitors to hospitalized patients in the neurology and nuclear medicine services. Patients and control subjects with a current or past medical condition or medication use that may influence bone mineral metabolism (hyperthyroidism, rheumatoid arthritis, hormone replacement therapy, use of oral contraceptives for a period longer that six months, past or recent use of hormone therapy, etc.) were excluded from the study. Patients and controls were asked to give 10 ml of blood for the study and they signed informed consent for testing. The research protocol and consent form was approved by the University Hospital of Ioannina Human Research Committee.

### Scanning

DXA scanning was performed utilizing a Hologic Discovery W scanner (Hologic, Bedford, MA, United States). All subjects had DXA scans performed in both lumbar spine (L1-L4) and left total hip in supine position. The scans and data analysis were performed by the same technician. For data analysis, a software package (v. 12.3:7, Hologic, Bedford, MA, United States) was employed as directed by the manufacturer. The measurements included the area of the examined bone, the bone mineral content (BMC) and the BMD of each anatomical site.

### Genotyping of Taq-I and Bsm-I polymorphisms

All molecular genetic studies were performed in the Laboratory of Medical Genetics in the Medical School of the University of Ioannina blinded to case control status. Genotyping of Taq-I (rs731236) and Bsm-I (rs1544410) was performed using TaqMan^® ^SNP Genotyping Assay which consists of a predesigned mix of unlabeled polymerase chain reaction (PCR) primers and the TaqMan^® ^minor groove binding group (MGB) probe (FAM™ and VIC^® ^dye-labeled). All TaqMan SNP Genotyping Assays are designed to work with TaqMan^® ^Universal PCR MasterMix which contains DNA polymerase, dNTPs and optimized mix components and uses the same thermal conditions. These assays were purchased from ABI (Applied Biosystems), and they were applied using a Real Time PCR Rotor-Gene RG3000 thermocycler (Corbett). The Taq1 T/C polymorphism is located in exon 9 that results in a Ile → Ile substitution for a silent codon change. It was genotyped using Real-Time PCR in which one probe labeled with VIC^® ^detects the T allele and the other probe labeled with FAM™ detects the C allele. Similarly, the Bsm1 G/A polymorphism at position 10,583,292 of the chromosomal region 12q12-q14 is located in the intron between exons 8 and 9 and is without consequences for the VDR protein structure. This SNP genotyped using VIC^® ^labeled probe for the G allele detection and FAM™ labeled probe for A allele detection, correspondingly. Real Time PCR was performed using 7.5 μl TaqMan^® ^Genotyping Master Mix (2×), 0.75 μl TaqMan^® ^SNP Genotyping Assay (TaqMan probes) (20×), 5.75 μl Dnase Free Water and 1 μl DNA (1-10 ng), to bring the final reaction volume to 15 μl. The Real Time PCR thermal conditions were as follows: Initial denaturing at 95°C for 10 min; 40 cycles of 96°C for 15 sec (denaturing) and 60°C for 1 min (annealing/extension). Samples were run in duplicates, with positive, negative controls and blanks. All materials were used in the TaqMan^® ^SNP Genotyping Assay (ABI) in accordance with the manufacturer's instructions and the information on the Applied Biosystems web site http://www.appliedbiosystems.com.

### Statistical analysis

Continuous data are presented as mean (standard deviation), categorical data as percentages. For comparisons of mean values between patients and controls statistical analysis was performed by the independent samples t-test. Categorical data was compared using comparison of two proportions (two-sided). Stepwise multiple regression analysis was performed to examine factors independently associated with the Taq-I and Bsm-I polymorphisms of the VDR gene. Gender, age, body mass index (BMI), MS diagnosis versus controls, smoking duration and lumbar spine/total hip BMD were included in the model as independent variables. P < = 0.05 was considered as statistically significant. The software used for the statistical analysis was STATISTICA (Stat Soft, Tulsa, OK).

## Results

The physical characteristics of the MS patients and control group are shown in Table 1. The characteristics are shown separately for females and males. The various parameters that were recorded include mean age, BMI, smoking years, BMD and expanded disability scale score (EDSS) and MS type for the patients.

**Table 1:** Physical characteristics of the MS patients (females and males) and control group [mean value ± standard deviation (range)].

	Females		Males	
		
Characteristics n	MS Patients (46)	Controls (58)	MS Patients (23)	Controls (23)
Mean age (years)	38.5 ± 9.9 (21-58)	38.6 ± 10.8 (20-60)	39.5 ± 11.8 (21-60)	38.8 ± 10.5 (23-60)
BMI (kg/m^2^)	24.9 ± 4.6 (16.8-34)	24.9 ± 5.1 (18.5-45.7)	25.1 ± 3.7 (17.4-36.4)	27.6 ± 3.2 (21.2-31.9)
Smoking period (years)	9.1 ± 10.8 (0-39)	8 ± 9.8 (0-33)	17 ± 12.4 (0-44)	12.4 ± 12.3 (0-38)
BMD (LS)	0.996 ± 0.16 (0.675-1.329)	1.025 ± 0.135 (0.738-1.29)	0.956 ± 0.12 (0.774-1.215)	1.028 ± 0.13 (0.71-1.26)
BMD (Hip)	0.857 ± 0.14 (0.542-1.132)	0.947 ± 0.123 (0.661-1.244)	0.915 ± 0.14 (0.615-1.286)	1.022 ± 0.1 (0.745-1.191)
EDSS	1.5 ± 1.8 (0 -5.5)		2.76 ± 2.5 (0 - 8)	
MS type	RRMS = 32		RRMS = 15	
	SPMS = 13		SPMS = 7	
	PPMS = 1		PPMS = 1	

MS: Multiple sclerosis; BMI: body mass index; EDSS: Expanded disability scale score; BMD: Bone mineral density; LS: Lumbar spine;

Hip: Total hip area; RRMS: Relapsing-remitting MS; SPMS: Secondary progressive MS; PPMS: Primary progressive MS.

The genotypic and allelic frequencies of the Taq-I (T > C) and Bsm-I (G > A) polymorphisms of the VDR gene are depicted in table 2. No statistically significant differences in the proportions of either polymorphism or allelic frequencies were found between the patient and the control groups. When the genotypic and allelic frequencies were compared between each MS subtype patient group (relapsing-remitting MS, secondary progressive MS and primary progressive MS) and control individuals, no statistically differences were observed as well (data not shown).

**Table 2:** Comparison between MS patients and control individuals of the Taq-I (T > C) and Bsm-I (G > A) polymorphisms of the VDR gene

Enzyme analysis n	Patients (69)	Controls (81)	p
Taq-I polymorphism			
Genotype			
TT	30 (43.5%)	33 (44.8%)	0.91
CT	30 (43.5%)	36 (44.4%)	0.94
CC	9 (13%)	12 (14.8%)	0.9
Allele			
T	65.2%	63%	0.78
C	34.8%	37%	0.80
Bsm-I polymorphism			
Genotype			
GG	28 (40%)	26 (32%)	0.54
GA	41 (60%)	55 (68%)	0.42
AA	0	0	
Allele			
G	70%	66%	0.60
A	30%	34%	0.6

MS: Multiple sclerosis; VDR: Vitamin D receptor.

Among the various parameters examined, statistically significant differences were found only in hip BMD between patients and control groups (Table 3). There was also a trend for lower BMD in the lumbar spine of the patients compared to controls but the result did not reach statistical significance (p = 0.06).

**Table 3:** Comparison of various parameters between MS patients and control individuals

Factor	Patients	Controls	p
Sex	46 Female	58 Female	
	23 Male	23 Male	
Mean age (years)	39.0 ± 10.5	38.7 ± 10.7	0.86
BMI (kg/m^2^)	24.8 ± 4.2	25.7 ± 4.8	0.23
Smoking period (years)	11.9 ± 11.8	9.3 ± 10.7	0.18
BMD (LS)	0.981 ± 0.15	1.025 ± 0.13	0.06
BMD (Hip)	0.875 ± 0.14	0.969 ± 0.12	<0.001*

BMI: body mass index; BMD: Bone mineral density; LS: Lumbar spine; Hip: Total hip area;

*: Statistically significant.

Multiple linear regression analysis of the Taq-I and Bsm-I genotypic frequencies showed independence from MS diagnosis versus controls, age, sex, smoking history, BMI, and lumbar spine or hip BMD (Table 4).

**Table 4:** Multiple linear regression analysis. Dependent variables Taq-I and Bsm-I (69 MS patients and 81 controls)

	Taq-I		Bsm-I	
Independent variable	Beta	p	Beta	p
MS versus controls	0.05	0.95	-0.04	0.62
Sex	-0.70	0.44	0.07	0.44
Age	-0.40	0.7	-0.11	0.32
BMI	0.05	0.63	0.00	0.99
Smoking years	0.12	0.19	-0.07	0.40
BMD (LS)	0.05	0.69	0.00	0.99
BMD (Hip)	-0.27	0.12	-0.29	0.20

BMD: bone mineral density; BMI: body mass index; MS: Multiple sclerosis; LS: Lumbar spine

## Discussion

In the present prospective study, we examined the Taq-I and Bsm-I common polymorphisms of the VDR gene, near its 3' untranslated region. Although these polymorphisms do not result in structural changes of the VDR protein, linkage disequilibrium with variation in the 3' untranslated region of the VDR gene may result in altered protein expression [18]. Our results showed no association between the Taq-I and Bsm-I polymorphisms of the VDR gene between the 69 MS patients and 81 control individuals, independently of the MS type.

Previous studies have shown either correlation or no association suggesting ethnic or regional differences [1,9]. For example, a Japanese group reported in 77 patients and 95 controls an overrepresentation of the Bsm-I G allele and homozygote GG in MS patients and in addition higher frequency of the Apa-I AA genotype and A allele in MS [10,19]. Similarly, an Australian case-control study in 104 MS patients and 104 controls, found a significant difference of genotype distribution between the case and control groups for the Taq-I polymorphism and the allelic frequency [11]. Partridge et al. in 419 cases and 422 controls described reduced expression of the Fok-I ff VDR genotype in MS patients [12]. It is interesting that the above studies that reported association of VDR gene polymorphisms and MS, involved populations with possibly high exposure to vitamin D either due to increased sunlight or higher intake.

Several large case-control studies have reported no association of the common VDR gene polymorphisms and MS risk. Thus, a US study in 214 MS cases and 428 age-matched controls found no associations for any of the single-nucleotide polymorphisms in VDR, but found an interaction between dietary intake of vitamin D and the VDR Fok-I polymorphism on MS risk. Specifically, individuals with the ff genotype had decreased MS risk with higher vitamin D intake [16]. Negative results have been reported in a Canadian study employed restriction fragment length polymorphisms and highly polymorphic microsatellite markers to study VDR gene polymorphisms and MS risk [15]. Α recent study in 136 MS cases and 235 controls showed no significant associations between the polymorphisms Cdx-2, Fok-I, or Taq-I and MS risk, but there was a significant correlation between winter sun exposure during childhood, the genotype at Cdx-2, and MS risk [13]. In that study the G allele was associated with increased risk of MS in the low sun exposure group (≤2 hours per day), indicating that an interaction of the VDR gene polymorphisms and MS may be dependent on past sun exposure [13]. Another study in 212 MS patients and 289 controls found no association of Apa-I and Taq-I VDR gene polymorphisms with the serum levels of 25(OH)D and 1,25(OH)(2)D, and MS risk [20]. A further study in the same cohort of patients, although failed to find any association of the Fok-I VDR gene polymorphism with MS, reported that the f-allele was associated with lower winter and summer serum 25(OH)D levels in MS patients and lower 25(OH)D levels in healthy controls. In addition, the f-allele carriers had higher 1,25(OH)(2)D/25(OH)D-ratios compared to f-allele carriers, indicating a possible role of the Fok-I VDR gene polymorphism in vitamin D metabolism [14]. Thus, certain polymorphisms of the VDR gene may have regulating effects on vitamin D function and metabolism and may be associated with MS risk, depending on previous or current vitamin D intake [9].

In the present study there was increased frequency of low BMD in MS patients compared to controls, especially involving the hip area and to a lesser degree the lumbar spine. Our results of low BMD in MS patients are in accordance with previous studies that demonstrated reduced BMD and increased frequency of osteoporosis in MS associated with low vitamin D levels [21,22]. However, the multivariate analysis failed to show any dependence of the low BMD from the Taq-I and Bsm-I polymorphisms of the VDR gene. Previous studies have showed no evidence of association between common polymorphisms of the VDR gene and BMD in British women [23], and between the Bsm-I VDR gene polymorphism and osteoporosis in a Korean population [24]. However, VDR gene polymorphisms have been associated with Graves' disease in a Japanese population [25], and has been suggested that a Fok-I VDR gene polymorphism may predict risk of osteoporosis in such patients [26].

## Conclusions

In summary, our findings showed no direct association of the Taq-I and Bsm-I polymorphisms of the VDR gene with either MS risk or bone mineral density in a sample population of Northwest Greece. Future studies should focus on the possible regulatory role of such polymorphisms on vitamin D function and metabolism and assess the MS risk depending on long term sunlight exposure and vitamin D intake.

## Competing interests

The authors declare that they have no competing interests.

## Authors' contributions

CS conceived of the study, participated in its design and wrote the manuscript. SP participated in the design of the study and in clinical data collection. SM participated in clinical data collection and the molecular genetic assays. FG performed the PCR assays for determination of the VDR polymorphisms. AG participated in the blood selection and determination of the BMD using DXA. IG participated in the design of the study, the genetic statistics and manuscript writing. SHP participated in the clinical data collection and study design. APK participated in the design of the study, data collection and coordination during writing of the manuscript. AF was responsible for the BMD determination using DXA in the laboratory of nuclear medicine and overview of the study. All authors read and approved the final manuscript.
